# Panretinal-Photocoagulation before Pars Plana Vitrectomy Influences Vitreous Level of Interleukin-6 but not of Vascular Endothelial Growth Factor in Patients with Diabetic Retinopathy

**Published:** 2007-03

**Authors:** Masahiko Shimura, Kanako Yasuda, Toru Nakazawa, Takashi Shiono, Kohji Nishida

**Affiliations:** 1 *Department of Ophthalmology, NTT East Japan Tohoku Hospital, Miyagi, Japan;*; 2 *Department of Ophthalmology and Medical Sciences, Tohoku University Graduate School of Medicine, Sendai, Japan;*; 3 *Shiono Eye Center, Sendai, Japan*

**Keywords:** diabetic retinopathy, interleukin-6 (IL-6), panretinal photocoagulation (PRP), pars plana vitrectomy (PPV), vascular endothelial growth factor (VEGF)

## Abstract

For eyes with diabetic retinopathy which require vitreous surgery and pan-retinal photocoagulation (PRP), pre-treatment of PRP before vitreous surgery reduce the activity of diabetic retinopathy, however sometimes cause macular edema leading to visual disturbance. Some cytokines in the vitreous increase in eyes with diabetic macular edema, thus the relationship between PRP and vitreous cytokines is to be investigated. In this study, 72 eligible eyes of 65 patients were recruited, and 36 eyes had pre-treatment of PRP before vitreous surgery. The other 36 eyes were served as control which had PRP not before but under surgery. There was no statistical significant difference of systemic conditions between two groups. All eyes had pars plana vitrectomy, and 1 ml of vitreous sample was obtained under the surgery. Vascular endothelial growth factor (VEGF) and interleukin-6 (IL-6) in the vitreous sample were measured in each case. After the completion of pre-treatment of PRP, macular edema defined as increase of foveal thickness was prominently worsened, and the vitreous level of IL-6 in PRP pre-treated group showed statistically higher than that in control. In contrast, there was no significant difference of VEGF level between two groups. While, vitreous level of VEGF in control group was strongly correlated with grade of retinopathy and duration of diabetes. In conclusion, PRP influenced vitreous level of IL-6 but not VEGF, leading to macular edema, which suggests that IL-6 plays critical roles of PRP induced macular edema.

## INTRODUCTION

Diabetic retinopathy is a leading cause of severe visual loss in advanced countries. It is estimated that 700000 Americans have proliferative diabetic retinopathy (PDR) with a projected incidence of 65000 new cases annually. Approximately 8000 new cases of blindness are caused by diabetic complications each year.

Pan-retinal scatter photocoagulation (PRP) is the established treatment in patients with PDR (1, 2). Despite the use of PRP to prevent proliferative retinopathy, PRP can sometimes cause macular edema which is recognized as the most common cause of vision decrease in diabetic eyes (3, 4). Earlier studies have shown that 25 to 43% of eyes with proliferative retinopathy treated by PRP develop macular edema and visual disturbances (5-8), however the detailed mechanisms of PRP induced macular edema remains unknown. Recent clinical studies showed that vitreous levels of cytokines, including interleukin-6 (IL-6), vascular endotheloial growth factor (VEGF) were increased in patients with diabetic macular edema(DME), and related to the severity of DME (9, 10). Also in animal studies, PRP induced upregulation of transforming growth factor beta2 (TGFβ2) (11), and increase of vitreous levels of IL-6, IL-8 and nitric oxide (NO) (12). Thus it is important to investigate the relationship between PRP-induced cytokines and PRP-induced macular edema.

Clinically, in front of patients with diabetic retinopathy who require vitreous surgery and PRP, vitreous surgeons sometimes meet the problem whether PRP should be done before or during vitreous surgery because PRP before surgery reduce the activity of diabetic retinopathy (13) but induce macular edema (5-8). In this study, vitreous samples were obtained at the time of pars plana vitrectomy (PPV) in patients with diabetic retinopathy with and without PRP and whether vitreous levels of IL-6 and VEGF were influenced by PRP 2 weeks before PPV were investigated.

## PATIENTS AND METHODS

### Patients Eligibility

In this prospective study, patients with diabetic retinopathy which require PPV and who have no history of retinal photocoagulation were recruited. The indications of PPV were as follows; existence of frequent vitreous hemorrhage (VH), proliferative tissue on the retina (PT), retinal detachment with traction (RD), diffuse retinal neovascularization (NV) and/or macular edema (ME). To estimate disease severity of diabetic retinopathy, each status (VH, PT, RD, NV, ME) scored 1 point, and the number of total score (1 to 5) defined the severity. Patients with renal dysfunction (BUN more than 20.0 mg/dl or creatinine more than 1.0 mg/dl) were excluded. Ophthalmological exclusion criteria were severe cataract, severe vitreous hemorrhage (impossible to observe the status of fundus by indirect ophthalmoscope) and severe macular edema (foveal thickness more than 450 μm).

Seventy two eyes of 65 patients (30 male, 35 female) participated in this study. The ages of the patients ranged from 55 to 78 years with a mean of 65.8 ± 5.8 years. All patients had type II diabetes, and the duration of the diabetes ranged from 2 to 15 years with a mean of 8.7 ± 2.5 years. At their initial examination, two blood pressure recordings were obtained with a mercury sphygmomanometer in sitting position after 10 minutes of rest and the averaged mean arterial pressure (MAP=2/3 (systolic blood pressure) + 1/3 (diastolic blood pressure)) was calculated. No history of any other ocular disease except refractive errors was reported. The HbA1c averaged 7.18 ± 0.96% (Table 1). All eligible eyes performed PPV and thirty-six eyes had been performed PRP before PPV. The other 36 eyes had not been done before PPV, of course in this control group, PRP was completed under PPV. Seven patients (4 male, 3 female) had bilateral eligible eyes, therefore pretreatment of PRP had done in one eye and the other eye defined as control. All patients received a comprehensive ocular examination before and periodically after the treatments. The systemic condition of all participants was monitored and treated appropriately by their internists for their general diabetic and hypertensive conditions.

**Table 1:** Clinical Charcteristics of the PDR Patinets who requires PPV

Characteristic	Pretreatment of PRP (n=36)	Control (n=36)	*p* value

Age (years)	66.4 ± 5.9	65.2 ± 5.8	0.35
Gender (male/female)	18/18	16/20	0.685
Duration of DM (years)	8.83 ± 3.53	8.47 ± 2.98	0.636
HbA1c (%)	7.12 ± 1.05	7.23 ± 0.87	0.377
Mean arterial pressure (mmHg)	98.75 ± 8.41	96.67 ± 9.27	0.248
Retinopathy grade	1.81 ± 0.86	1.81 ± 0.95	0.844
Initial foveal thickness (μm)	361.0 ± 5.5	374.7 ± 41.7	0.359

After informing the patient of the purpose of this study and the possible outcomes, informed consent was obtained from all patients prior to the interventions. This study was also approved by the NTT East Japan Tohoku Hospital Clinical Research Ethics Committee. The procedures were performed to conform to the tenets of the Declaration of Helsinki.

### Experimental Design

In this study, to investigate the effect of PRP before PPV, pre-treatment of PRP was performed 2 times at 1 week interval and 1 more week after that (2 weeks after the beginning of PRP), PPV was performed in treated eyes. In control eyes, instead of pre-treatment of PRP, PRP was performed during PPV. At the beginning of PRP (0 week) and just before PPV (2 week), foveal thickness (FT) was measured using optical coherence tomography (OCT: Zeiss-Humphrey model OCT-3000, Dublin, CA). A macular thickness map was constructed from six radial scans that intersected at the fovea using the OCT retinal mapping program (version 6.2). This program calculates mean thickness in nine regions: the 1000 μm central area and the four quadrants of an inner and outer ring. The inner ring had an inner diameter of 1000 μm and an outer diameter of 3000 μm, and the outer ring had an inner diameter of 3000 μm and an outer diameter of 6000 μm (14, 15). In this study, FT was defined as the mean value within a 1000 μm central area and its value reflected macular edema.

### Panretinal Scatter Photocoagulation

Because the PRP scatter treatment was designed to be completed in two sessions, the order of the treated area in the fundus was inferior hemisphere first, and then superior area. The size of the spots on the retina was 200-500 μm, and the duration of the application was 0.15-0.2 second with the Volk Super Quad 160 fundus laser lens [Volk] and a krypton red laser [Nidek, JAPAN] mounted on a slit-lamp [Zeiss, Germany] The number of spots in each session was around 700-800, thus the total number of burns after the four sessions was about 1500. Topical anesthesia was used in all cases, and all patients were treated as outpatients. In control group, PRP was completed with endophotocoagulation probe [Novus Spectra, LUMENIS, Tokyo, Japan] under PPV. The conditions of photocoagulation were also 200-500 μm of spots size on the retina, 0.2 second of duration, and about 1500 spots of the number in each case.

### Measurement of IL-6 and VEGF levels

Samples of vitreous fluid were collected into sterile tubes at the time of vitrectomy and rapidly frozen at -80°C IL-6 levels in vitreous samples were measured using chemiluminescent enzyme immunoassay (CLEIA) kits for human IL-6 (Fuji Rebio Co, Ltd. Tokyo, Japan). Assays were performed according to the manufacturer’s instructions. In brief, 50 μl of the standard solution and the sample were put into the activation cartridge for 10 min, 37°C. After incubation, the samples were rinsed and an enzyme-labeled antibody was added. After further incubation, the samples were rinsed again and the 3-(2’-spiroadamantane)-4-methoxy-4-(3”-phosphoryloxy) Phenyl-1, 2-dioxetane disodium salt (AMPPD) was added. The reaction was stopped, and the optical density was determined using a photo-counting luminometer (Lumipulse 1200, Fuji Rebio Co, Ltd.). A standard curve was plotted from measurements made with the standard solution (0.3-300 pg/ml, and was used to determine the concentration of IL-6 in the sample. The minimal detectable concentration was 0.3 pg/ml (intra-assay coefficient of variation [CV]=2.8%; interassay CV=5.6%).

VEGF levels in vitreous sample were measured using enzyme-linked immunosorbent assay (ELISA) kits for human VEGF (Quantikine^®^ R&D Systems, Minneapolis, MN). Assays were performed according to the manufacturer’s instructions. The VEGF kit detected the two short, secreted VEGF isoforms (VEGF121 and VEGF165) but not the the two longer, cell-associated isoforms. One hundred μl of the standard solution and the sample were added to the wells of a 96-well plate coated with a monoclonal antibody. After incubation, the samples were rinsed and an enzyme-labeled antibody was added. After further incubation, the samples were rinsed again and the substrate was added. The reaction was stopped after color developed, and the optical density was determined at 450 and 630 nm using a microplate reader (Vmax, Molecular Device Japan, Tokyo Japan). A standard curve was plotted from measurements made with the standard solution (15.6-1000 pg/ml) and was used to determine the concentration of VEGF in the sample. The minimal detectable concentration was 15.6 pg/ml (intra-assay CV=5.4%; interassay CV=7.3%).

### Statistical analysis

Because the obtained data do not always follow a metric scale, the significant differences between the preand post-treatment data in the same group was assessed by the Wilcoxon signed-rank test, and between the treated-and control eyes was done by the Mann Whitney U test as appropriate using a statistical program [SPSS Science, Chicago, IL]. To investigate the correlation between vitreous cytokine levels and clinical parameters (sex, patient’s age, duration of DM, grade of retinopathy, MAP and FT), pearson’s correlation coefficient (r), and a p value was calculated. Since the number of samples was 36, a |r| value of more than 0.3 was considered statistically significant, and |r|<0.6 defined as a strong correlation. All of the data are presented as means ± standard deviations.

## RESULTS

### Patients’ characteristics between PRP treated- and control eyes

In this study, although 7 subjects had bilateral eligible eyes, the other 58 subjects had unilateral; therefore patients’ characteristics between two groups should be clarified. As shown in Table 1, there is no statistical significant difference of patient’s age (*p*=0.350), gender (*p*=0.685), duration of diabetes (*p*=0.636), HbA1c (*p*=0.377), and MAP (*p*=0.248). Also, there is no statistical significant difference of severity of diabetic retinopathy (*p*=0.844), and initial FT (*p*=0.359). These results indicate that clinical conditions in both groups could be the same before starting this study, and it is suitable for comparative study. During PRP before PPV (2 weeks after the beginning of PRP), the status of the retinopathy was not dramatically changed in all cases.

### PRP affects foveal thicknes

**Figure 1:**
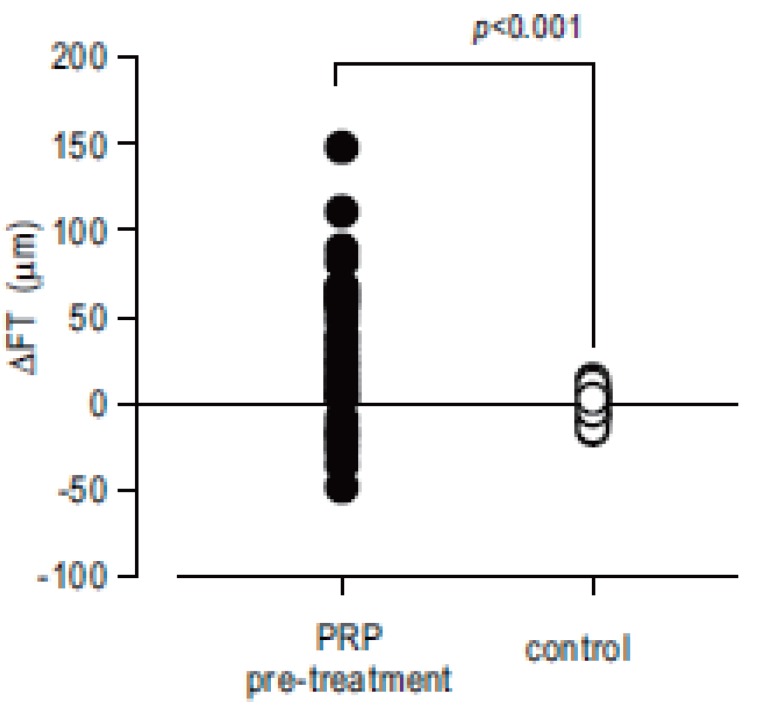
The delta FT (ΔFT) defined as subtraction of FT 2 wks after the initial exam from FT at the initial exam was calculated. ΔFT in PRP pre-treated group (●) showed statistically significant more increase than that in control groups (○) (*p*<0.001).

In PRP pre-treated group, FT was increased from 361.0 ± 55.5 μm to 392.8 ± 67.9 μm (*p*=0.799) after the completion of PRP (2 week after the initial exam), in contrast FT in control eye was not altered from 374.7 ± 41.7 μm to 375.1 ± 40.3 μm (*p*<0.001) during 2 weeks before PPV. The delta FT (ΔFT) defined as subtraction of FT 2wks after the initial exam from FT at the initial exam was calculated in each groups, and ΔFT in PRP pre-treated group showed statistically significant more increase than control groups (*p*<0.001) (Fig. 1) Interestingly, 4 of 36 eyes with pretreatment of PRP showed more than 5% decrease of FT.

### PRP affects vitreous level of VEGF and IL-6

**Figure 2:**
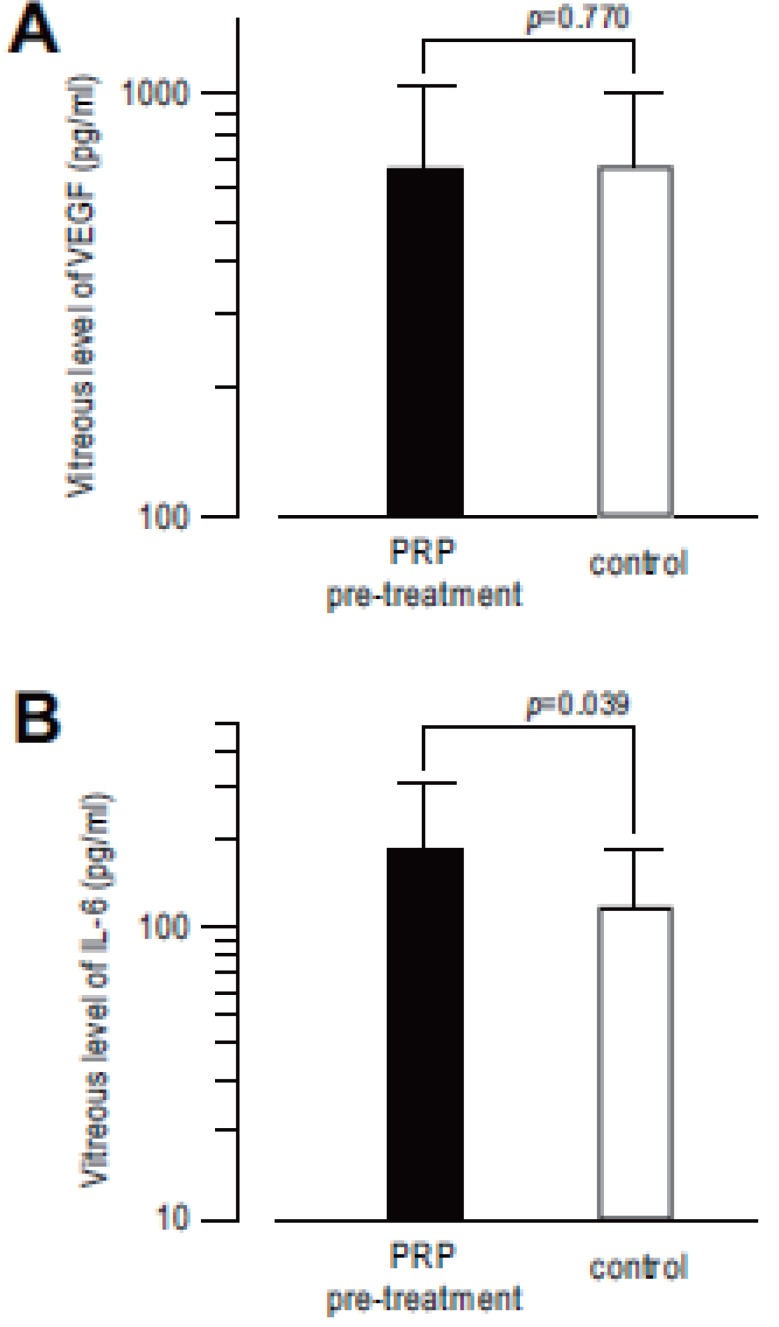
Vascular endothelial growth factor (VEGF) (A) and Interleukin-6 (IL-6) (B) concentrations in the vitreous fluid of PRP pre-treated eyes and control eyes in patients with diabetic retinopathy which requires pars plana vitrectomy. There is no statistical significant difference of vitreous level of VEGF between two groups (A, p=0.770), in contrast, vitreous level of IL-6 in PRP pre-treated group is statistically higher than that in control group (B, *p*=0.039).

Vitreous level of VEGF was 661.1 ± 373.5 pg/ml (ranging 112 to 1379 pg/ml) in PRP pre-treated eyes, and 667.7 ± 341.4 pg/ml (ranging 165 to 1374 pg/ml) in control eyes. There is no statistical significant difference between two groups (*p*=0.769). (Fig. 2A) In contrast, vitreous level of IL-6 in PRP pre-treated group (183.5 ± 126.2 pg/ml (ranging 27.5 to 437 pg/ml)) shows statistically significant higher than that in control (115.7 ± 68.1 pg/ml (ranging 23.7 to 283 pg/ml) (*p*=0.038)) (Fig. 2B). From these results, vitreous level of IL-6 was significantly increased 2 weeks after PRP, but that of VEGF did not affected by PRP.

### The relationship between cytokines and clinical parameters

To investigate the relationship between vitreous levels of cytokines (VEGF and IL-6) and each clinical factor (patient’s age, gender, duration of diabetes, HbA1c, MAP, severity of diabetic retinopathy, initial FT, pre-operative FT and ΔFT), correlation coefficient was calculated (Table 2). In PRP pre-treated group, both VEGF and IL-6 was correlated with ΔFT only, while in control group, VEGF was statistically correlated with duration of diabetes and strongly with grade of retinopathy and IL-6 was strongly correlated with HbA1c.

**Table 2:** Correlation coefficient between vitreous cytokines and clinical factors

	Correlation coefficient between VEGF and clinical factors				Correlation coefficient between IL-6 and clinical factors			
	PRP pre-treated		Control		PRP pre-treated		Control	

Clinical factor	*r*	*p*	*r*	*p*	*r*	*p*	*r*	*p*

Patients’ age	-0.026	0.883	-0.089	0.606	0.072	0.681	-0.103	0.552
Gender	-0.121	0.489	0.096	0.582	0.043	0.803	-0.231	0.179
Duration of diabetes	0.173	0.316	0.403	0.014	0.159	0.356	0.146	0.398
HbA1c	0.302	0.073	-0.104	0.551	0.049	0.777	0.929	<0.001
MAP	0.261	0.125	0.045	0.796	0.111	0.524	0.219	0.202
Grade of retinopathy	0.283	0.095	0.772	<0.001	0.213	0.215	0.042	0.809
Initial FT	-0.091	0.599	-0.294	0.082	0.033	0.852	-0.015	0.932
Delta FT	0.552	0.001	0.055	0.752	0.647	<0.001	0.116	0.502

PRP, panretinal photocoagulation; *r,* correration coefficient; *p*, level of significance; MAP, mean arterial pressure; FT, foveal thickness.

### Comparison in bilateral cases

As described before, there were seven patients who had bilateral eligible eyes and one eye was PRP pre-treated and the other eye was served as control. In these bilateral cases, it is possible to exclude the effects which influenced by systemic conditions. As shown in Table 3, between pre-treated and control eyes, there is no significant difference of severity of retinopathy (*p*<0.001) and initial foveal thickness (*p*=0.499) by Wilcoxon signed rank test. Even in these bilateral case-control series of 14 eyes in 7 patients, Vitreous level of IL-6 in PRP pre-treated eyes was more significantly increased than that in control eyes (*p*=0.018), in contrast there is no statistical significant difference of vitreous level of VEGF between PRP pre-treated and control eyes (*p*=0.735). These results confirm that pre-treatment of PRP before PPV influences vitreous level of IL-6 but not of VEGF in patients with diabetic retinopathy.

**Table 3:** Comparison in bilateral cases

patient #	age (years)	duration (years)	HbA1c (%)	MAP (mmHg)	

**Patients characteristics**					
1	56	10	8.3	105	
2	78	11	7.6	94	
3	64	3	7.2	111	
4	58	14	8.1	93	
5	74	9	6.3	90	
6	60	7	7	96	
7	63	9	7.4	90	
Mean ± SD	64.7 ± 8.26	9.00 ± 3.42	7.41 ± 0.68	97.0 ± 8.0	
**patient #**	**grade**	**VEGF (pg/ml)**	**IL-6 (pg/ml)**	**initial FT (μm)**	**ΔFT (μm)**

**PRP treated eyes**					
1	1	694	142	340	30
2	2	993	180	298	67
3	1	192	128.8	360	-48
4	1	417	45.4	384	4
5	1	440	203	337	17
6	2	369	279	418	12
7	1	502	78.1	420	63
Mean ± SD	1.286 ± 0.488	515.3 ± 258.7	150.9 ± 78.5	365.3 ± 44.9	20.7 ± 39.0
**Control eyes**					
1	1	856	140	338	2
2	3	916	71.7	302	-2
3	1	283	115	356	14
4	1	403	29.1	391	1
5	1	427	176.7	345	-5
6	1	276	90.2	425	3
7	1	591	52.9	412	-7
Mean ± D	1.286 ± .756	536.0 ± 61.7	96.5 ± 1.3	367.0 ± 4.0	0.86 ± .87

## DISCUSSIONS

Along with medical progression of preventive care for diabetes mellitus, retinal photocoagulation against diabetic retinopathy has been widely performed in advanced countries. Pan-retinal scatter photocoagulation (PRP) is the established treatment in patients with PDR (1, 2), but sometimes brings decreased visual acuity due to PRP-induced macular edema (3, 4, 16-18) which could be related with vitreous cytokines including VEGF and IL-6 (9). Because only human and monkey have “macula” in retina, it is difficult to investigate the pathogenesis of PRP induced macular edema. In this clinical study, it was possible to evaluate the status of macular thickening with the use of OCT retinal imaging, and also possible to evaluate the influence of PRP to vitreous cytokines by obtaining vitreous samples from eyes with diabetic retinopathy which require PPV.

According to previous studies, both VEGF and IL-6 in vitreous fluid was increased in patients with diabetic macular edema (9, 10). From this study, it was clearly showed that PRP accelerated the diabetic macular thickening and increased vitreous level of IL-6, indicating that IL-6 plays critical role of PRP induced macular edema. Interestingly, vitreous level of VEGF, which is a key factor of progression of retinopathy (19) did not induced by PRP. IL-6 is an inflammatory cytokine. In contrast, VEGF is an endothelial specific mitogen that can induce endothelial permeability (20), and its production is upregulated by ischemia (21). It is reasonable that vitreous levels of both cytokines are increased in diabetic macular edema (9, 10) because there is chronic inflammation with hyperpermeability in retinal vessels (including vascular endothel) of patients with diabetes mellitus (22). However PRP cause acute thermal destruction of retinal tissue, which is likely to be associated with inflammation. Thus, the inflammatory cytokine IL-6 can cause endothelial barrier dysfunction (23) and increase endothelial permeability (20), leading to macular edema. As for VEGF, chronic damage in retinal vessels might lead to retinal tissue hypoxia, and induce VEGF expression (21) and VEGF increases endothelial permeability by loosening tight junctions (24, 25), therefore it is not surprising that VEGF is not soon influenced by acute injury of retinal photocoagulation. Also, there are some evidences that IL-6 indirectly induces VEGF *in vitro* (26, 27) and our results that vitreous level of VEGF was well correlated with duration of diabetes and grade of retinopathy in control eyes support the hypothesis that PRP-induced expression of VEGF was much slower than that of IL-6. Other possible explanation was that vitreous level of VEGF in diabetic patients had already saturated before this study, and thus PRP did not influence its expression. Further investigation should be done to clarify the mechanism of different expression of IL-6 and VEGF induced by PRP. Of course VEGF and IL-6 were not only responsible factors of diabetic retinopathy, therefore, other reported intravitreous cytokines/chemokines including ICAM (10), SDF-1 (28), or polyamines including spermidine, putrescine, and spermine (29), should be studied in future.

After the completion of PRP, FT in PRP treated group showed significant increase, however 4 of 36 eyes with PRP showed more than 5% decrease of FT. vitreous level of VEGF in these 4 eyes was 264.3 ± 187.2 pg/ml (112, 192, 216, 537 pg/ml) which is lower than those in the other 32 eyes. PRP does not always induce macular edema and reduces the activity of diabetic retinopathy (13), and ΔFT in PRP treated eyes was statistically correlated with VEGF (r=0.552, *p*=0.0004) and IL-6 (r=0.647, *p*<0.0001). Therefore in the diabetic eyes with lower cytokines in the vitreous, PRP predominantly reduce the activity of retinopathy, leading regression of macular edema.

For vitreous surgeons, it still remains to be debated whether pre-treatment of PRP should be done before PPV in patients with diabetic retinopathy. Clinically we recognized that PRP under PPV had not always induced macular edema, but PPV without pretreatment of PPV sometimes cause surgical complications, including vitreous hemorrhage and/or retinal detachment under PPV. In this case-control study, there were no severe surgical complications under PPV in control (without pretreatment of PPV) as well as in PRP pre-treated group, thus it seems to be recommended that PRP should be done not before but under PPV. However, to confirm this hypothesis, visual prognosis after PPV must be required. In this study, it is hard to estimate visual prognosis because some patients had visual disturbance not only by retinopathy but lens opacity (cataract). A larger and longer study should be necessary to solve the question that “for patients with diabetic retinopathy who requires PPV, PPV should be done after the completion of PRP or PRP should be completed under PPV?”.

## PROPRIETARY INTEREST

The author declares that no conflicting interests exist.
